# A case report of congenital idiopathic hypogonadotropic hypogonadism caused by novel mutation of *GNRHR* gene

**DOI:** 10.1097/MD.0000000000024007

**Published:** 2021-02-05

**Authors:** Liping Wang, Weisheng Lin, Xiaohong Li, Lijuan Zhang, Kai Wang, Xiaoli Cui, Shanmei Tang, Guangguang Fang, Yan Tan, Xuelai Wang, Chuan Chen, Chuanchun Yang, Huiru Tang

**Affiliations:** a Peking University Shenzhen Hospital; b Shenzhen Key Laboratory of Gynecological Diagnostic Technology Research; c CheerLand Precision Biomed Co., Ltd.; d Shenzhen Dapeng New District Maternity & Child Health Hospital Department of Gynecology; e Shenzhen Second People's Hospital, The First Affiliated Hospital of Shenzhen University, Shenzhen, China; f Li Ka Shing Faculty of Medicine, School of Biomedical Science, the University of Hong Kong, Hong Kong.

**Keywords:** congenital idiopathic hypogonadotropic hypogonadism, *GNRHR*, missense mutation, novel mutation

## Abstract

**Rationale:**

This study aimed to investigate the genetic mutation characteristics of congenital idiopathic hypogonadotropic hypogonadism (IHH) through the clinical features and genetic analysis of 2 patients with IHH in 1 pedigree.

**Patient concerns:**

A 23-year-old girl presented with primary amenorrhea, sparse pubic hair, lack of breast development, and delayed sexual development.

**Diagnoses:**

Combined with the clinical characteristics, auxiliary examinations, and molecular genetic analysis, the patient was diagnosed as IHH.

**Interventions:**

Whole exome and Sanger sequencing were performed to validate the mutation in family members.

**Outcomes:**

A novel homozygous missense mutation c.521A > G (p.Q174R) in the *GNRHR* gene was identified in the 2 affected sisters. Familial segregation showed that the homozygous variant was inherited from their parents respectively and the eldest sister was the carrier without correlative symptom.

**Lessons:**

We reported a novel *GNRHR* mutation in a pedigree with congenital idiopathic hypogonadotropic hypogonadism. Glutamine at amino acid position 174 was highly conserved among various species. The molecular structure of *GNRHR* protein showed that p.Q174R mutation brought in a new stable hydrogen bond between position 174 and 215, may impede conformational mobility of the TMD4 and TMD5. It suggests that the missense mutation c.521A > G related to congenital idiopathic hypogonadotropic hypogonadism was probably a causative factor for both sisters. Through high-throughput sequencing and experimental verification, we had basically determined the patient's pathogenic mutation and inheritance, which could better guide doctors for treatment.

## 1 Introduction

Congenital idiopathic hypogonadotropic hypogonadism (IHH) is a rare genetic form of hypogonadism, characterized by delayed or absent puberty. Other associated nonreproductive phenotypes, such as cleft palate, sensorineural hearing loss, and anosmia, occur with variable frequency.^[1]^ IHH is termed as Kallmann syndrome, when in the presence of anosmia or hyposmia, or referred to as normosmic IHH (nIHH), without olfactory abnormalities.^[2]^ Up to now, related phenotypic variability and its genetic heterogeneity have been described.^[3]^ The occurrence of IHH in men is more common than women, with a male predominance of 3–5:1.^[4]^

*GNRHR* is the first gene reported to be associated with nIHH,^[5]^ located on chromosome 4q13. It encodes the GnRH receptor, comprising 3 coding exons, and contains 7 transmembrane domains, inducing LH and FSH secretion.^[6]^ Hypogonadotropic hypogonadism-7 with or without anosmia is caused by homozygous or compound heterozygous mutations in the *GNRHR* gene.^[7,8]^ Interestingly, female patients of nIHH due to biallelic *GNRHR* mutations have been scarcely reported, but some cases are exceptions.^[9,10]^ Women with nIHH/bi-*GNRHR* showed variable puberty, but nearly all present with primary amenorrhea.^[10]^

## 2 Methods

Under the premise of informed consent, we performed whole exome sequencing^[1]^ for the proband, using genomic DNA from peripheral blood. WES library preparation was captured with a biotinylated oligonucleotides probes library (Agilent SureSelect Human All Exon v.6, Agilent), subsequently sequenced on an Illumina HiSeq X-Ten platform (Illumina Inc., San Diego, CA). The sequencing raw data were collected, then the adapter sequence was removed, and the low quality reads were discarded. The filtered data were aligned to the human genome reference assembly (UCSC Genome Browser hg19; https://genome.ucsc.edu/index.html) with the Burrows-Wheeler Aligner, and the variants were called by GATK. All variants were annotated by ANNOVAR. Functional annotation information included 1000 Genomes Project (1000G), Exome Aggregation Consortium (ExAC), Genome Aggregation Database (gnomAD), OMIM, ClinVar, and Human Gene Mutation Database (HGMD). The impact of the sequence variants on protein function was evaluated with SIFT, PolyPhen-2, Revel, and Mutation Taster.

To detect and determine the mutation identified by WES, polymerase chain reaction (PCR) and Sanger sequencing were conducted in family members. The *GNRHR* coding region sequence was investigated through NCBI GenBank, and by using Primer 5.0 software (London, Ontario, Canada) to design the primers of the *GNRHR* gene. Primers were as follows: *GNRHR*-F: CCACTGGATGGGATGTGGAA; and *GNRHR*-R: AGGTCTTATCAAAGGAAGTACTGT. PCR amplification was utilized under the following conditions: 92 °C for 30 seconds, 32 cycles of 92 °C for 30 seconds, 55 °C for 30 seconds, 70 °C for 30 seconds, and 70 °C for 10 minutes. The purified PCR products were sent for Sanger sequencing (Sangon Biotech, Shanghai, China). The sequencing results were then analyzed using Chromas software and aligned against the *GNRHR* (NM_000406) sequence shown in the NCBI database.

## 3 Case report

**Figure 1:**
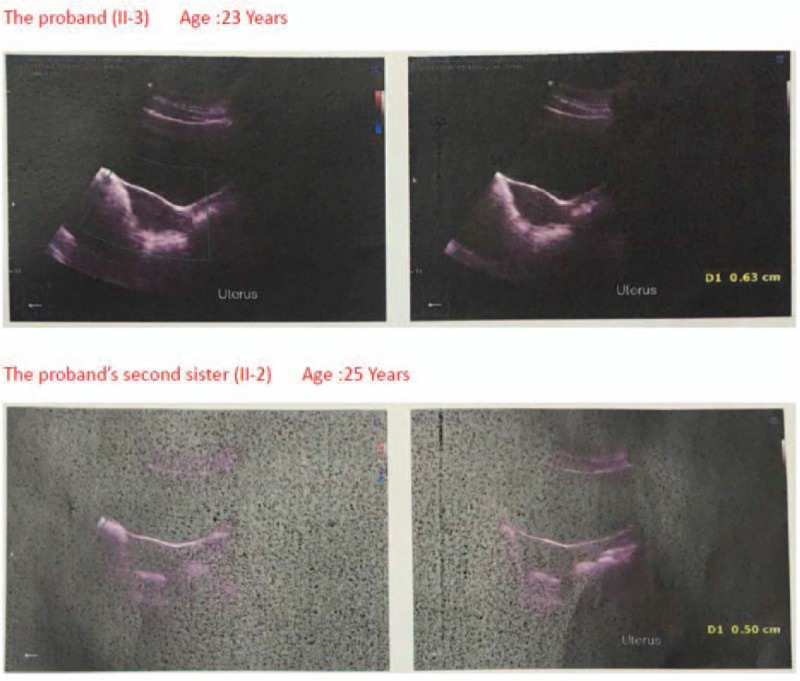
Ultrasound image of patients: proband and her second eldest sister both appeared small uterus.

**Figure 2:**
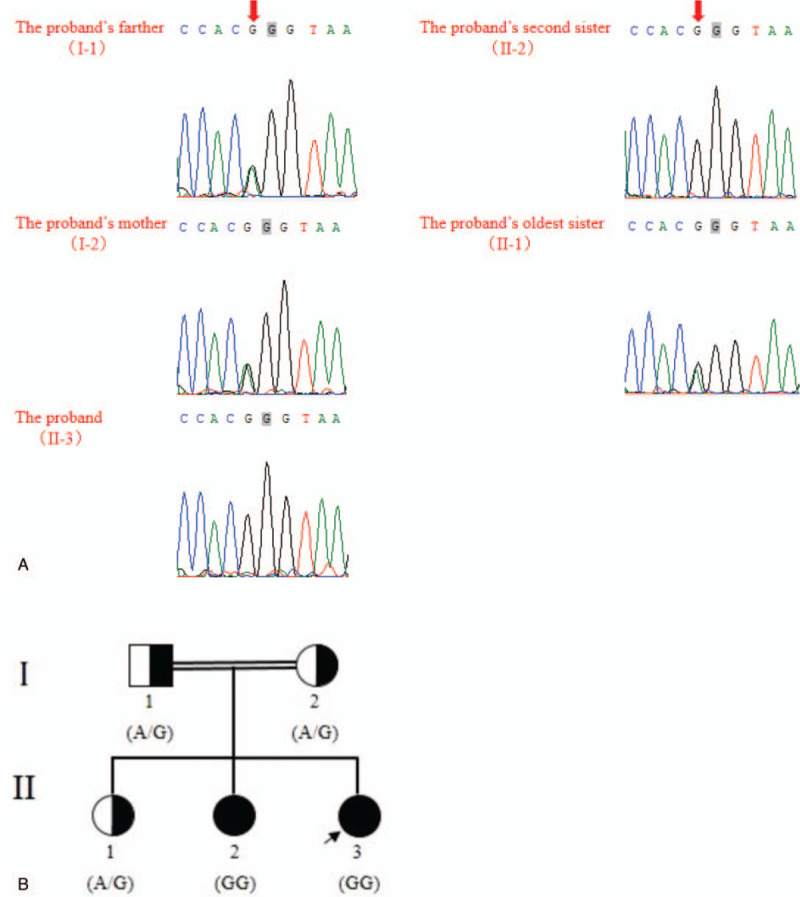
Mutation in sisters with IHH. (A) Sanger sequencing confirmed the mutation c.521A > G in *GNRHR.* The proband and her second eldest sister were homozygote, whereas her parents and eldest sister were heterozygote. The arrows indicated the position of mutation c.521A > G. (B) Pedigrees affected by IHH. The affected members (proband and her second eldest sister were indicated as black circles). IHH = idiopathic hypogonadotropic hypogonadism.

We studied a consanguineous Chinese family. The proband was a 23-year-old girl. She and her 25-year-old sister had primary amenorrhea, sparse pubic hair, lack of breast development, and delayed sexual development, which were suspected as congenital idiopathic hypogonadotropic hypogonadism. On blood tests, the 2 sisters had a very low level of serum luteinizing hormone (LH) and follicle-stimulating hormone (FSH) (Table 1). The ultrasound image showed that both the 2 sisters had small uterus (Fig. 1A). No record of significant illnesses was discovered in them. Their parents were cousins (Fig. 2B). By WES and Sanger sequencing, a novel homozygous mutation in *GNRHR* (NM_000406) c.521A > G:p.Q174R was confirmed in the affected sister and the parents as well. The other sisters were carriers with the heterozygous state (Fig. 2A). Through high-throughput sequencing and experimental verification, we had basically determined the patient's pathogenic mutation and inheritance, which could better guide doctors to use drugs to relieve the symptoms.

**Table 1:** Hormone test in patients.

Patient	Age, y	FSH, IU/L	LH, IU/L
Proband	15	0.60	0.13
	18	0.50	0.20
Proband's second eldest sister	16	0.60	0.33
	20	0.30	<0.10

**Figure 3:**
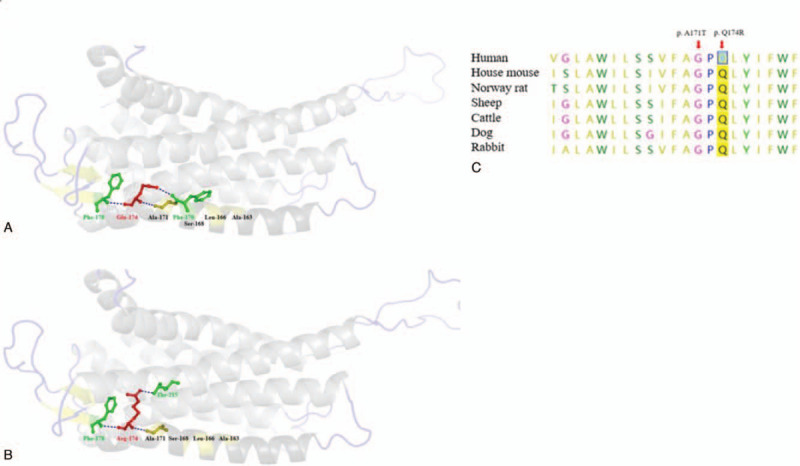
Structural model and conservation of mutation in *GNRHR.* (A) and (B) Three-dimensional structure of GNRHR, showing the protein domain. The positions of Ala163, Leu166, Ser168, Ala171, with olive color, were recorded in HGMD, located on the TMH4. The position of Q174 was on TMH4 too. (A) The structure of wild type, showing the Gln174 (Q174) with red color, connected with Phe170, Ala171, and Phe178 by hydrogen bond. (B) The structure of mutation, showing the Arg174 (R174) with red color, brought in a newly stable hydrogen bond between position Arg174 on TMH4 and Thr215 on TMH5. (C) The conservation of the p.Q174R and p.A171T in *GNRHR* was analyzed among various species. HGMD = Human Gene Mutation Database.

This mutant was absent in the 1000 Genomes Project and ExAC, and presents in heterozygous state in the gnomAD, with an allele frequency of 0.000008202, but not in the homozygous state. To date, the missense homozygous mutation detected in this study has never been reported as pathogenic in the literature or various databases. The effect of protein function for this novel mutant predicted all were deleterious. To further investigate the mutation p.Q174R, we used multiple sequences alignment and molecular modeling analyses to assess the effect on the protein. Glutamine at amino acid position 174 and its nearby residues were highly conserved among various species (Fig. 3C). The mutation p.Q174R locates on the TMD4 of the GnRH receptor on the base of the Pfam database. Furthermore, we discovered that the wild-type Gln174 connected with Phe178, Ala171, and Phe170 by hydrogen bond on the TMD4 (Fig. 3A). Yet, the mutant residue Arg174 was predicted that without joint to Phe170, leads to connection with Thr215 on TMD5 (Fig. 3B). Therefore, we concluded that this mutation may destroy the conformational mobility of TMD4 and TMD5.

Based on the results, we suggest that the patients need to undergo genetic counseling and preimplantation genetic testing for aneuploidy (PGT-A) before giving birth to avoid the risk of the disease.

## 4 Discussion

Congenital idiopathic hypogonadotropic hypogonadism (IHH) is rarely a genetic hypogonadism, and it occurs in men more commonly than women.^[2]^ Few women cases about nIIHH due to biallelic *GNRHR* mutations have been reported. Diagnosis is based on the occurrence of low levels of sex hormones associated with inappropriate or low FSH and LH levels, with no other pituitary hormone deficiencies, and no anatomical lesion in the hypothalamic–pituitary tract.^[11]^ Genetic studies of patients with IHH have identified oligogenic and monogenic defects in several genes that regulate the synthesis, secretion or action of GnRH, or the embryonic development or migration of GnRH neurons.^[12]^ The pedigree reported in this study may be a further expansion of IHH female cases.

In this study, we identified a homozygous mutation c.521A > G at exon 1 of *GNRHR* gene in the 2 affected sisters with nIHH from a consanguineous family. Our study indicates that this novel mutant may be one of the genetic causes of congenital idiopathic hypogonadotropic hypogonadism.

The *GNRHR* gene is related to hypogonadotropic hypogonadism, encoding the GnRH receptor. And the GnRH receptor regulates LH and FSH secretion, containing 7 transmembrane domains (TMD). The candidate variant p.Q174R is located on the TMD4 of the GnRH receptor. Meanwhile, there are 4 variations (p.A163A, p.L166P, p.S168R, and p.A171T) on the TMD4, identified in the affected individuals with hypogonadotropic hypogonadism.^[13–16]^ Especially, the p.Ala171Thr mutation has been reported in 2 brothers with severe hypogonadotropic hypogonadism, and the vitro expression confirmed that it may play an important role in signal transduction.^[17]^ We believed that the p.Q174R mutation may prevent the signal from transmitting intracellularly. To elucidate the deleterious effect of p.Q174R mutation, we built a three-dimensional structure for both wild-type and the candidate variant by Swiss-Model (Fig. 3A and B). The result showed that the mutation p.Q174R led to the introduction of a newly stable hydrogen bond between position 174 and 215, which may impede conformational mobility of the TMD4 and TMD5 (Fig. 3A and B). Interestingly, multiple sequence alignment indicated that the position substitution of GnRH receptor was highly conserved among various species (Fig. 3C).

Moreover, this novel homozygous mutant was absent in the 1000 Genomes Project and ExAC, recorded at gnomAD with an allele frequency of 0.000008202, but not in the homozygous state. Simultaneously, SIFT, PolyPhen-2, and Mutation Taster all predicted that this mutation was deleterious. At the same time, Sanger sequencing indicated the proband and her second eldest sister had homozygous state, and her eldest sister as well as her parents were heterozygous (Fig. 2A). Taken together, we deduce that the mutation of *GNRHR* may be the genetic cause of nIHH. Nevertheless, this needs further research.

## Acknowledgments

The authors thank Peking University Shenzhen Hospital for providing samples and ethical approval and Technology-CheerLand Institute of Precision Medicine for providing research equipment.

## Author contributions

**Formal analysis:** Guangguang Fang, Xuelai Wang, Chuan Chen.

**Funding acquisition:** Huiru Tang.

**Investigation:** Liping Wang, Xiaohong Li.

**Methodology:** Kai Wang, Xiaoli Cui, Shanmei Tang.

**Supervision:** Yan Tan, Chuanchun Yang, Huiru Tang.

**Writing – original draft:** Weisheng Lin, Lijuan Zhang.

**Writing – review & editing:** Weisheng Lin.
